# Human umbilical cord mesenchymal stem cells for psoriasis: a phase 1/2a, single-arm study

**DOI:** 10.1038/s41392-022-01059-y

**Published:** 2022-08-05

**Authors:** Lamei Cheng, Siqi Wang, Cong Peng, Xiao Zou, Chao Yang, Hua Mei, Chuang Li, Xian Su, Na Xiao, Qi Ouyang, Mi Zhang, Qiaolin Wang, Yan Luo, Minxue Shen, Qun Qin, Honglin Wang, Wu Zhu, Guangxiu Lu, Ge Lin, Yehong Kuang, Xiang Chen

**Affiliations:** 1 Institute of Reproductive and Stem Cell Engineering, School of Basic Medical Science, Central South University, Changsha, 410031 China; 2 National Engineering Research Center of Human Stem Cell, Changsha, 410205 China; 3 The Department of Dermatology, Xiangya Hospital, Central South University, Changsha, 410008 China; 4 Hunan Engineering Research Center of Skin Health and Disease, Hunan Key Laboratory of Skin Cancer and Psoriasis, National Clinical Research Center for Geriatric Disorders (Xiangya Hospital), Changsha, 410008 China; 5 The Office of Drug Clinical Trials Institution, Xiangya Hospital, Central South University, Changsha, 410031 China; 6 Precision Research Center for Refractory Diseases,Shanghai General Hospital, Key Laboratory of Cell Differentiation and Apoptosis of Chinese, Ministry of Education, Shanghai Jiao Tong University School of Medicine, Shanghai, 200025 China; 7 Reproductive and Genetic Hospital of CITIC-Xiangya, Changsha, 410221 China

**Keywords:** Mesenchymal stem cells, Immunological disorders, Clinical trials

## Abstract

Psoriasis is a common, chronic immune-mediated systemic disease that had no effective and durable treatment. Mesenchymal stem cells (MSCs) have immunomodulatory properties. Therefore, we performed a phase 1/2a, single-arm clinical trial to evaluate the safety and efficacy of human umbilical cord-derived MSCs (UMSCs) in the treatment of psoriasis and to preliminarily explore the possible mechanisms. Seventeen patients with psoriasis were enrolled and received UMSC infusions. Adverse events, laboratory parameters, PASI, and PGA were analyzed. We did not observe obvious side effects during the treatment and 6-month follow-up. A total of 47.1% (8/17) of the psoriasis patients had at least 40% improvement in the PASI score, and 17.6% (3/17) had no sign of disease or minimal disease based on the PGA score. And the efficiency was 25% (2/8) for males and 66.7% (6/9) for females. After UMSC transplantation (UMSCT), the frequencies of Tregs and CD4^+^ memory T cells were significantly increased, and the frequencies of T helper (Th) 17 and CD4^+^ naive T cells were significantly decreased in peripheral blood (PB) of psoriasis patients. And all responders showed significant increases in Tregs and CD4^+^ memory T cells, and significant decreases in Th17 cells and serum IL-17 level after UMSCT. And baseline level of Tregs in responders were significantly lower than those in nonresponders. In conclusion, allogeneic UMSCT is safe and partially effective in psoriasis patients, and level of Tregs may be used as a potent biomarker to predict the clinical efficacy of UMSCT. Trial registration Clinical Trials NCT03765957

## Introduction

Psoriasis is a common, chronic immune-mediated systemic disease determined by polygenic inheritance and induced by multiple environmental factors. The prevalence of psoriasis varies from 0.27% to 11.43% of the world’s population.^1^ In China, psoriasis has an estimated prevalence of 0.47%, affecting more than 7 million patients.^2^ Psoriasis is a disease with innate and adaptive immune system disorder and dendritic cells, neutrophils, keratinocytes and T cells play major roles in the pathogenesis. When genetically susceptible individuals are exposed to infection, stress or trauma, cutaneous dendritic cells are activated to produce tumor necrosis factor (TNF)-α and interleukin (IL)-23, which subsequently stimulate the proliferation and differentiation of proinflammatory T cells. The proinflammatory T cells secrete inflammatory cytokines, including interferon (IFN)-γ and IL-17, which affect keratinocytes and other leukocytes that forms an amplified immune response.^3^ Immunological and genetic studies have identified IL-17/IL-23 axis as a key driver of psoriasis pathogenesis.^4^ Biologic agents targeting TNF-α, IL-17 and IL-23 have been developed and achieved great success.^3^ However, patients treated with biologic agents are prone to relapse, and adverse events, such as nasopharyngitis, upper respiratory tract infection and tuberculosis may occur.^5^ Therefore, it is urgent to find safer therapeutic approaches for psoriasis.

MSCs are multipotent cells characterized by their regenerative capabilities and immunomodulatory properties. It is well established that MSCs possess immunomodulatory functions on T cells, B cells and other innate immune cells, mainly through cell-to-cell contacts^6^ or paracrine mechanisms by secreting extracellular vesicles and cytokines, including transforming growth factor-β (TGF-β), nitric oxide (NO), indoleamine-pyrrole 2,3-dioxygenase (IDO), tumor necrosis factor-stimulated gene-6 (TSG-6), prostaglandin E2 (PGE2) and IL-10.^7–9^ In vitro, when co-cultured with PBMC, MSCs were able to secret PGE2 to inhibit the proliferation, activation and differentiation of Th1 and Th17 cells^10^ and secret TGF-β to up-regulate the percentage of Tregs.^11^ The regulatory effect of MSCs on T cells has also been demonstrated in mice models of autoimmune disease such as rheumatoid arthritis and sjogren syndrome (SS). MSCs could decrease the number of Th1 cells and increase the number of Tregs in draining lymph nodes (DLNs),^12^ increase the percentage of Th2 cells and reduce the frequency of Th17 cells in spleen,^13^ and increase the level of IL-10 in joints^12^ and salivary gland.^13^ Furthermore, MSC-derived extracellular vesicles also exert their immunomodulatory properties through the transfer of RNAs and proteins.^14^ In an imiquimod (IMQ)-induced psoriasis mice model, subcutaneous injection of MSCs drastically diminished the severity of dorsal lesions by decreasing the percentage of IL-17-producing γδ T cells in DLNs and declining the level of IL-17 in skin lesions.^3^ Our previous studies showed that intravenous infusion of UMSCs could also significantly reduce disease severity, skin inflammatory cell infiltration and serum cytokine production in IMQ-induced mice models of psoriasis through regulating the balance of Th1, Th2, and Th17 responses in both the spleen and DLN, suppressing the function of neutrophils and down-regulating the production of IFN-α by plasmacytoid dendritic cells.^15^

The immunomodulatory ability of MSCs makes them rational candidates for the treatment of autoimmune disease in clinical researches. A recent study of MSCs transplantation for type 1 diabetes (T1D) demonstrated that fasting C-peptide levels was improved and insulin requirement was reduced, and companied with an increase in the level of IL-10 and a decrease in IFN-γ in serum of T1D patients after MSC treatment.^16^ It was reported that after MSC treatment, the serum levels of IL-1β, IL-6, IL-8, and TNF-α in rheumatoid arthritis patients were reduced, IL-10 level was increased and the 28-joint disease activity score was significantly decreased.^17^ Also, a multicenter clinical study of MSCT in active and refractory SLE illustrated that MSCs could significantly decline disease activity and improve renal function as well as serologic indices.^18^ The underlying therapeutic mechanisms may be that MSCs could up-regulate Tregs proportion and decrease the percentage of Th17 cells by secreting TGF-β and PGE2.^11^ There have been several case reports of MSCs treatment for psoriasis which showed certain therapeutic effects.^19,20^ Autologous adipose-derived MSCs were used for one patient with psoriasis vulgaris and the other with psoriatic arthritis. The PASI of the patients decreased (from 21.6 to 9.0, 24.0 to 8.3, respectively) after several MSC infusions, and no serious adverse events were noted.^21^ Two cases of psoriasis vulgaris treated by umbilical cord-derived MSCs showed that both patients maintained a four- or five-year relapse-free period.^22^

In this study, we performed a phase 1/2a, single-arm study to evaluate the safety and efficacy of UMSC infusions in the treatment of psoriasis. We analyzed the changes in immune cell subsets and serum cytokines to explore the possible mechanism of action of UMSCs.

## Results

### Patient Characteristics

**Fig. 1:**
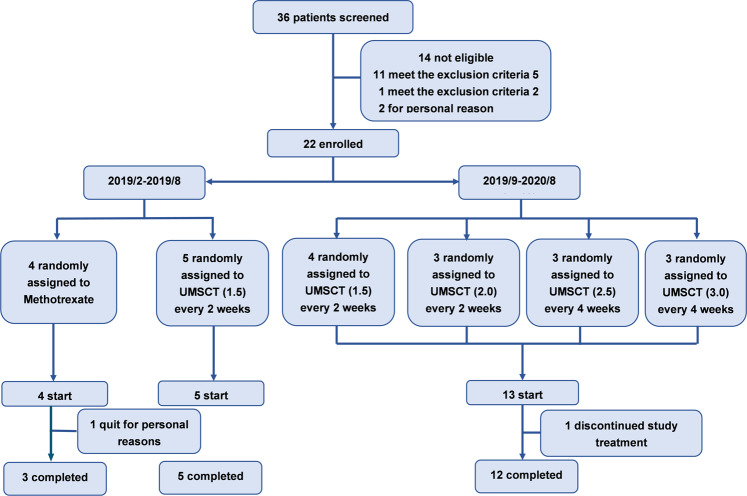
Flowchart of the study

Thirty-two patients were included between March 2019 and August 2020. Fourteen patients were not eligible for meeting the exclusion criteria and personal reasons. A total of 18 patients were enrolled and received UMSCT, of which 17 patients completed UMSC infusions according to the clinical protocol, and 1 patient discontinued the treatment (Fig. 1). The study was divided in 2 stages. The first stage was from Feb to Sep 2019, 5 subjects were enrolled and received UMSC infusions once every 2 weeks in a single dose of 1.5 × 10^6^/kg, 4 times as a course of treatment. In the second stage, from Sep 2019 to Aug 2020, 13 patients were enrolled, 1 patient discontinued because of worsening of psoriasis, and 12 completed the study. 3 patients in each group. Patients were heterogeneous in terms of age (29–55 years), disease duration (4–32 years), body mass index (20.2–31.2), and PASI score (10.8–30.7) at baseline. Five patients had a family history of psoriasis. The vast majority of patients had used topical agents previously, and 11.8% had used biologic agents. All patients were dissatisfied with or intolerance of the efficiency of topical agents. The patients who received biological agents had participated in the clinical trial of our hospital, and the treatment was effective but relapsed after the end of the clinical trial. The baseline demographic and disease characteristics of the patients are summarized in Table 1.

**Table 1:** Baseline characteristics of the patients

Patient number	Sex/Age	Body weight (kg)	Body mass index	Duration of psoriasis (yr)	Baseline PASI score (%)	Baseline BSA (%)	Baseline PGA	UMSC Dose/kg (×106)	Previous treatment
2019/2–2019/8 (*n* = 5)									
S01	F/32	54	20.3	25	30.7	34.6	5	1.5	MTX, CsA, AcI, PUVA
S02	M/55	61	22.6	10	26.6	32.8	5	1.5	AcI, IL-17 monoclonal antibody, TA
S03	M/29	82	26.8	14	20.4	42.0	4	1.5	TCM, PUVA, TA
S04	F/39	51	20.2	27	25.6	67.0	5	1.5	MTX, AcI, PUVA, levocetirizine, Yisaipu
S05	M/44	76	26.3	9	17.1	17.5	4	1.5	TCM
2019/9–2020/8 (*n* = 12)									
S09	F/50	70	26	32	13.5	19.0	4	1.5	MTX, TCM, PUVA, TA
S10	M/53	67	23.2	9	15.2	20.0	4	1.5	TCM, TA
S11	M/55	60	20.8	30	13.2	12.3	3	1.5	MTX, Folic acid, levocetirizine, Ssg, TA
S12	F/32	61	24.4	19	11.3	18.1	4	2.0	TCM, tirixizine, TA
S13	M/34	63	23.1	20	10.8	12.9	4	2.0	TCM, TA
S14	F/30	55	20.8	7	16.9	28.8	4	2.0	Ssg, TCM, TA
S15	F/39	62	25.1	10	24.4	32.0	5	2.5	AcI-A, TA
S16	M/46	76	31.2	20	23.8	51.1	4	2.5	AcI, Ssg, TA
S17	F/37	50	20.8	7	13.4	12.9	4	2.5	AcI, TA
S18	F/31	51	21.2	10	16.4	15.1	4	3.0	Ssg, TA
S19	F/48	80	27.3	16	15.4	13.8	4	3.0	CsA, AcI, TA
S20	M/35	66	22.5	4	15.0	12.6	4	3.0	Ssg, PUVA, TA

*PASI* psoriasis area and severity index, *BSA* body surface area, *PGA* physicians’ global assessment; Only systemic previous therapies: *MTX* methotrexate, *CsA* cyclosporin, *AcI* Acitretin; *TA* Topical agents, *TCM* Traditional Chinese medicine; *Ssg* Silver shavings granule

### Safety

There were no serious adverse events associated with UMSC infusion. Three patients developed low fever immediately after infusion, which resolved within 24 h with symptomatic treatment. Another 2 patients developed dizziness and chills that resolved within 24 h after resting; 1 of them developed an elevated myocardial enzyme profile and electrocardiography abnormalities, but no abnormalities were observed in subsequent monitoring. Other side effects that may be associated with infusion, such as elevated skin temperature and inflammation, were resolved after symptomatic treatment. Importantly, we did not observe major abnormalities pre- and post-UMSCT in the following parameters: liver functional parameters (including aspartate aminotransferase, alanine transaminase and bilirubin); renal function parameters (including serum creatinine, serum urea and uric acid); myocardial zymogram (including lactate dehydrogenase, creatine kinase and its isoenzymes, myoglobin); glucose and lipid metabolism (including fasting blood glucose, triglyceride, cholesterol); or systemic inflammation and other safety parameters (such as C-reactive protein, ESR, immunoglobulin IgA/IgM/IgG). No obvious abnormalities were observed in chest X-ray and ECG, and no infection complications or cancer were found until 6 months of follow-up. However, blood tests showed that the PB leukocyte count, neutrophil count, urea nitrogen and myoglobin decreased significantly after UMSCT at month 6, but all values were within the normal range (Table 2). No safety or tolerability concern was identified up to a maximum single 3.0 × 10^6^/kg IV dose of UMSCs.

**Table 2:** Safety evaluation on patients pre- and post- UMSCT

Measures (normal value range)	Psoriasis		*p* value
	Pre-UMSCT	Post-UMSCT	
White blood cell count (3.5–9.5 × 10^9^/L)	7.38 (0.40)	6.38 (0.39)**	0.003
Neutrophils count (1.8–6.3 × 10^9^/L)	4.80 (0.29)	3.99 (0.26)**	0.008
Lymphocyte count (1.1–3.2 × 10^9^/L)	1.70 (1.35,2.25)	1.60 (1.20,1.85)	0.360
Platelet count (125–350 × 10^12^/L)	276.00 (236.00,320.00)	253.00 (240.00,296.00)	0.356
Alanine aminotransferase (9.0–50.0 U/L)	18.80 (12.40,30.55)	16.70 (12.55,27.90)	0.740
Aspartate aminotransferase (15.0–40.0 U/L)	19.80 (18.40,25.05)	19.80 (16.20,24.75)	0.523
Blood urea nitrogen (3.10–8.00 mmol/L)	4.14 (0.26)	3.67 (0.22)*	0.017
Uric acid (155–357 mmol/L)	323.60 (24.95)	322.24 (20.19)	0.920
Creatinine (41–111 μmol/L)	73.75 (2.99)	72.49 (3.06)	0.251
Creatine Kinase (50–310 U/L)	84.00 (58.50,111.50)	75.50 (64.25,102.25)	0.619
Lactate dehydrogenase (120–250 U/L)	180.00 (165.00,191.25)	166.00 (146.25,181.60)	0.136
Myoglobin (<70 μg/L)	28.30 (21.25,32.25)	21.70 (19.00,27.40)*	0.030
Fasting blood glucose (3.90–6.10 mmol/L)	5.67 (5.15,6.10)	5.32 (4.88,5.98)	0.143
Cholesterol (<5.18 mmol/L)	4.89 (0.15)	4.92 (0.13)	0.860
Complement C4 (100–400 mg/l)	264.06 (16.92)	242.47 (11.93)	0.066
Complement C3 (790–1250 mg/L)	967.24 (27.86)	934.29 (28.10)	0.210
IgG (7–16 g/L)	12.43 (0.54)	11.80 (0.49)*	0.038
IgA (700–5000 mg/L)	2629.24 (257.01)	2338.47 (275.03)	0.202
IgM (400–2800 mg/L)	969.00 (748.00,1250.00)	867.00 (720.00,1285.00)	0.163
C-reactive protein (0–8 mg/L)	1.62 (0.26)	1.90 (0.19)	0.323
Erythrocyte sedimentation rate (0–26 mm/h)	18.00 (15.00,38.50)	14.00 (10.00,28.00)	0.214

Psoriasis *n* = 17, Data are median (IQR) or mean (SD), IQR interquartile range, *P <0.05, **P < 0.01, Pre- vs Post-UMSCT

### Efficacy

**Fig. 2:**
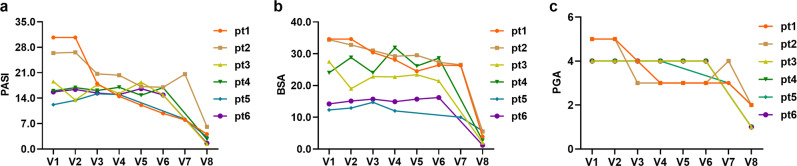
The figure showed three outcome measures of the 6 responders including PASI score, BSA and PGA. **a** PASI score; **b** BSA; **c** PGA

**Fig. 3:**
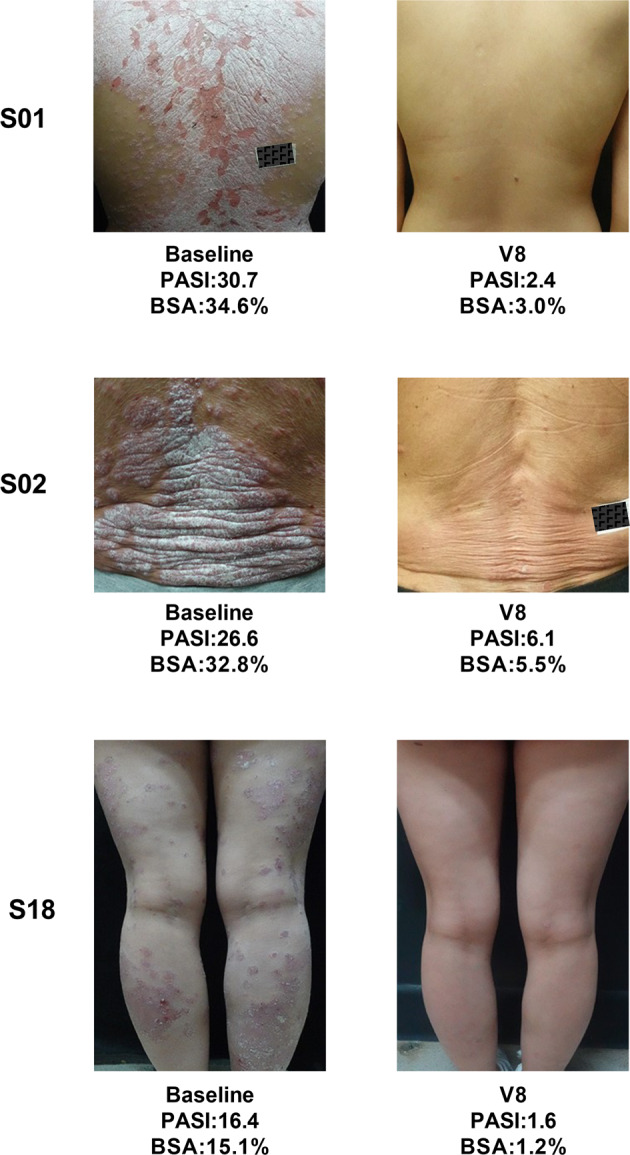
Representative skin images of 3 patients before and after treatment

At the sixth month, among the patients who received UMSCT, 47.1% (8/17) had at least 40% improvement, 35.3% (6/17) had more than 75% improvement (Fig. 2) and 17.6% (3/17) had more than 90% improvement in the PASI score. See the representative images of patients in Fig. 3. However, the remaining 9 patients did not show significant improvement. Three patients showed no sign of disease (a score of 0) or minimal disease (a score of 1) based on the PGA score. Furthermore, we found that the efficiency of female patients (66.7%, 6/9) was higher than that of male patients (25%, 2/8), and we did not find significant differences in other aspects, such as the severity of the disease, between female and male patients (Table 3). Due to the limitation of the small sample size, it is too early to draw conclusions, but it is worth further phase 2 clinical study with a larger sample size to confirm whether there is indeed a gender difference in MSC treatment of psoriasis.

**Table 3:** Comparison of baseline characteristics between male and female psoriasis patients

	Female	Male	*p* value
*N*	9	8	
Mean age (y)	37.56 (2.44)	43.88 (3.62)	0.160
Body Mass Index	22.90 (0.92)	24.56 (1.19)	0.280
Duration of psoriasis (y)	17.00 (3.10)	14.50 (2.96)	0.571
PASI	18.62 (2.22)	17.76 (1.92)	0.776
BSA	18.75 (12.68,39.70)	19.00 (14.45,33.30)	0.727
PGA	4.00 (4.00,4.00)	4.00 (4.00,5.00)	0.250
Efficiency	6/9 (66.7%)	2/8 (25%)	

Data are median (IQR), *n* (%), or mean (SD), IQR: interquartile range, Female vs Male

### Changes in immunity

We analyzed the immune-related changes in the PB of patients pre- and post-UMSCT by multiparameter flow cytometry (Supplementary Table 1).

**Fig. 4:**
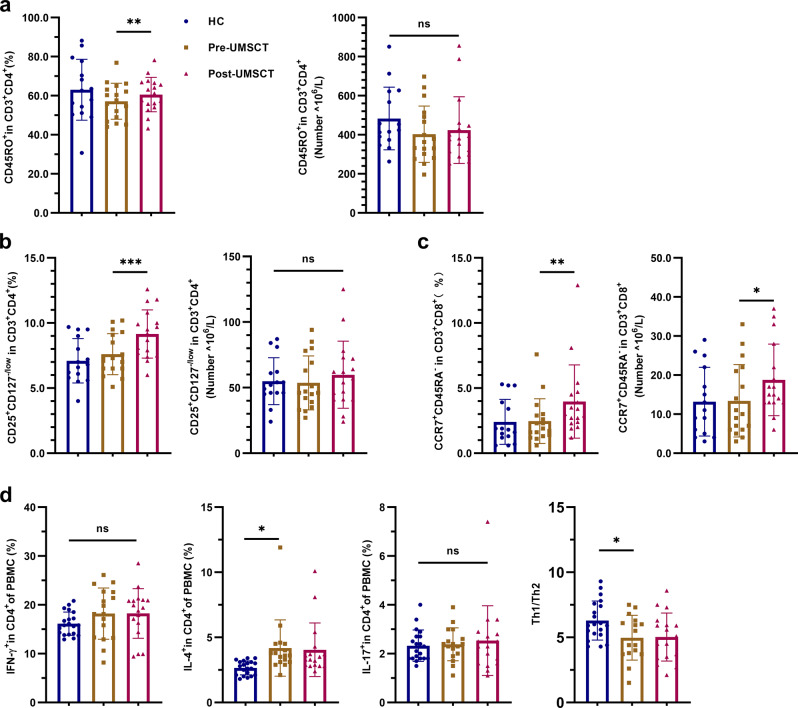
Frequencies and numbers of T lymphocyte subsets in PB of HC and patients with psoriasis pre- and post-UMSCT. **a** Frequencies and numbers of CD4^+^ memory T cells (CD45RO^+^ in CD3^+^CD4^+^). **b** Frequencies and numbers of Tregs (CD25^+^CD127^-^/low in CD3^+^CD4^+^). **c** Frequencies and numbers of CD8^+^ TCM cells (CCR7^+^CD45RA^-^ in CD3^+^CD8^+^). (HC, *n* = 15; Patients, *n* = 17; ns, no significant, VS HC; *P < 0.05, **P < 0.01, ***P < 0.001 VS Pre-UMSCT); **d** Frequencies of Th1, Th2, Th17 and the ratio of Th1/Th2. (HC, *n* = 15; Patients, *n* = 17; *P < 0.05, VS HC; ns, no significant, VS Pre-UMSCT

First, we analyzed the T lymphocyte subsets of the psoriasis patients and healthy controls (HCs). We found that the proportion of IL-4^+^ in CD4^+^ (Th2) cells was significantly higher in psoriasis patients than in HCs. However, the proportions of IFN-γ^+^ in CD4^+^ (Th1) and IL-17^+^ in CD4^+^ (Th17) cells did not show significant differences between psoriasis patients and HC (Fig. 4d), as did the proportions and the numbers of CD45RO^+^ in CD3^+^CD4^+^ (CD4^+^ memory T), CD25^+^CD127^−/low^ in CD3^+^CD4^+^ (Treg) and CCR7^+^CD45RA^-^ in CD3^+^CD8^+^ (CD8^+^ TCM) cells (Fig. 4a–c). Second, we analyzed the changes in T lymphocyte subsets in psoriasis patients pre- and post-UMSCT. The results showed that the frequencies of CD45RO^+^ in CD3^+^CD4^+^ (CD4^+^ memory T), CD25^+^CD127^−/low^ in CD3^+^CD4^+^ (Treg) and CCR7^+^CD45RA^−^ in CD3^+^CD8^+^ (CD8^+^ TCM) cells were significantly increased after UMSCT. However, we did not observe significant changes in the numbers of these cells after UMSCT (Fig. 4a–c).

**Fig. 5:**
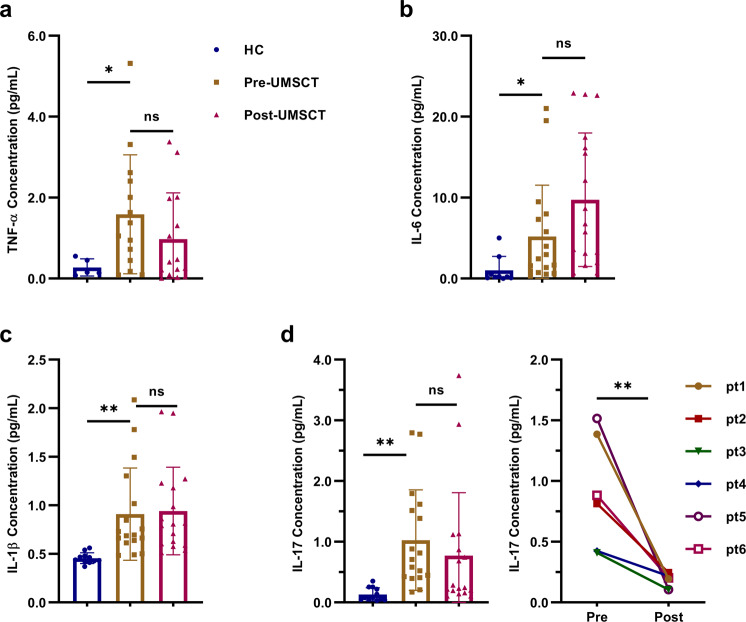
The inflammatory factors in the serum in HC and psoriasis patients pre- and post-UMSCT. **a** Serum TNF-α concentration. **b** Serum IL-6 concentration. **c** Serum IL-1β concentration. **d** Serum IL-17 concentration in HC, all psoriasis patients and responders pre- and post-UMSCT. (*n* = 17, **P < 0.01, ***P < 0.001, vs Pre-UMSCT)

Analysis of the inflammatory factors in the serum showed that the levels of TNF-α, IL-6, IL-1β, and IL-17 were significantly higher in psoriasis patients than in HCs (Fig. 5a–d). However, we did not observe significant changes in these cytokines in psoriasis patients pre- and post-UMSCT.

**Fig. 6:**
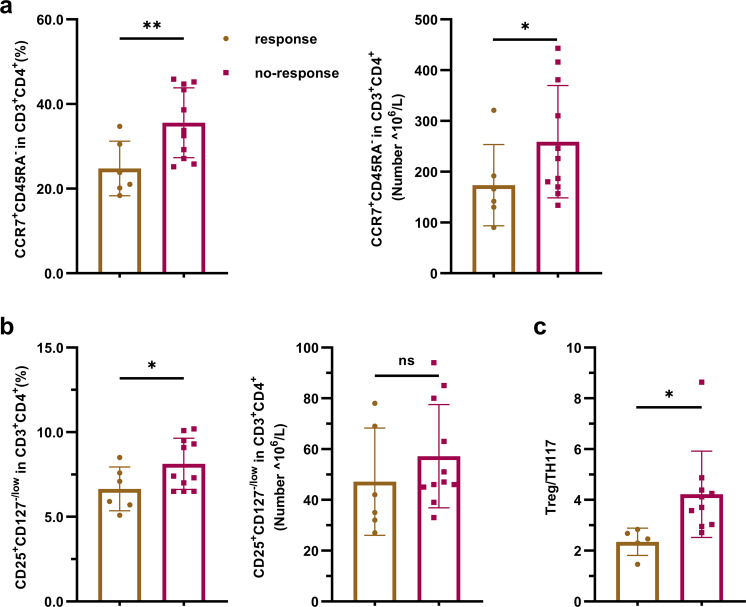
Baseline immune indices in PB in response group and no-response group. **a** Baseline level of CD4+TCM cells. **b** Baseline level of Tregs. **c** Baseline level of the ratio of Treg/Th17 (response group: *n* = 6, no-response group: *n* = 11, *P < 0.05; **P < 0.01)

**Fig. 7:**
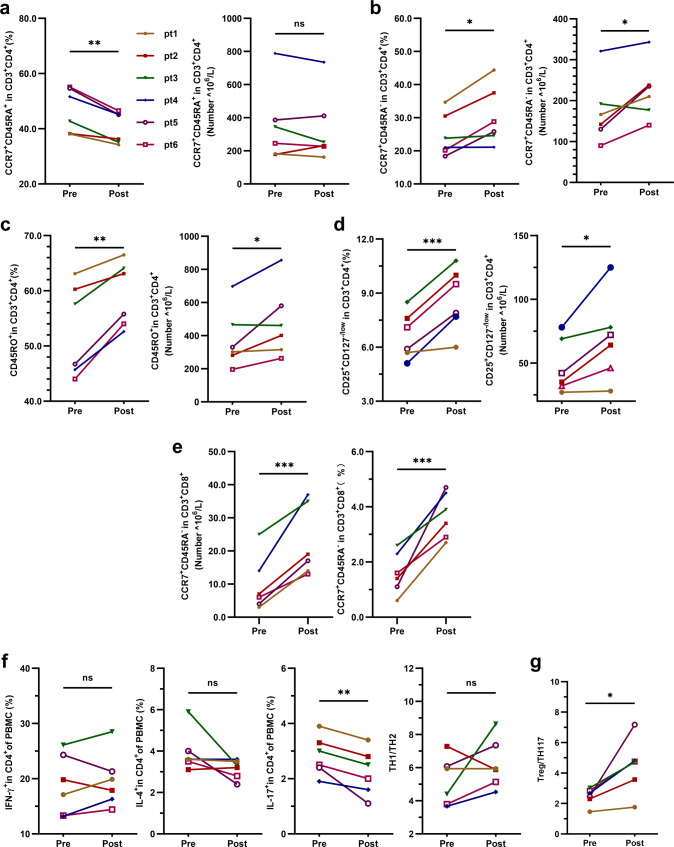
The changes in T lymphocyte subsets in responders pre- and post-UMSCT. **a**–**e** Changes of frequencies and numbers of CD4 naive T, CD4+ TCM, CD4+ memory T, Treg and CD8+ TCM cells in PB in responders (*n* = 6, *P < 0.05, **P < 0.01, ***P < 0.001); **f**−**g** Changes of frequencies of Th1, Th2, Th17 and Th1/Th2 and Treg/Th17 in PB in responders (*n* = 6, *P < 0.05, **P < 0.01)

Based on the aforementioned results, we tried to analyze the factors related to the effectiveness of UMSC treatment. Patients were divided into a response group (responders: PASI improvement ≥75%, *n* = 6) and a no-response group (nonresponders: PASI improvement<75%, *n* = 11; Supplementary Fig. 1) according to their degree of improvement. Baseline immune indices were compared between these two groups. The results revealed that the levels of CD4^+^ TCM cells, Treg cells and Treg/Th17 cells in the response group were significantly lower than those in the no-response group (Fig. 6a–d). We also analyzed the changes in T lymphocyte subsets in responders pre- and post-UMSCT and found that the frequencies of CD4^+^ naive T cells and Th17 were significantly decreased (Fig. 7a, f), the frequencies and numbers of CD4^+^ TCM, CD4^+^ memory T cells, Treg and CD8^+^ TCM cells were significantly increased after UMSCT (Fig. 7b–e), and the ratio of Treg/Th17 was significantly increased in responders (Fig. 7g). These results suggested that UMSCs might play an immunoregulatory role by promoting the differentiation of naive T cells into effector T cells and memory T cells. Furthermore, we found that the levels of IL-17 in responder serum were significantly decreased after UMSCT (Fig. 5d), which suggested that UMSCs may exert a therapeutic effect by reducing the level of serum IL-17, which is consistent with the increase in Treg/Th17 cells.

Moreover, significant changes in the numbers and frequencies of other immune cells, such as some T lymphocyte subsets, NK cells and B-cell subsets, were not observed between HC and psoriasis patients or psoriasis pre- and post-UMSCT. (Supplementary Figs. 2–5).

## Discussion

To our knowledge, only limited clinical investigations have been conducted on MSC treatment in patients with psoriasis. We previously conducted a preclinical study of the intravenous delivery of UMSCs to treat imiquimod (IMQ)-induced mouse models of psoriasis. In this study, the phase 1/2a clinical trial demonstrated that the application of clinical-grade UMSCs is safe and partly effective in patients with psoriasis, and the efficiency appears to be related to sex, with this treatment being more effective in women than in men. However, due to the small sample size, it is necessary to further expand the sample size to confirm the result.

With the main objective of our trial, no serious adverse events or major abnormalities in clinical analysis or biochemical indices for heart, liver and kidney function were observed, and no infection complications or cancer were observed during UMSCT or follow-up. Although decreased PB leukocyte counts and neutrophil counts were observed in patients who received UMSCT at the 6-month follow-up, the changes were negligible, and no related adverse events were found during the study. Our study set different single infusion doses of 1.5, 2.0, 2.5, and 3.0 × 10^6^/kg. Patients who received infusions of UMSC with cell numbers up to 2.4 × 10^8^ (3.0 × 10^6^/kg) also showed no obvious side effects after infusions, such as fever and thrombus, or adverse effects throughout the study, especially in vital parameters and routine blood tests. However, we cannot conduct dose climbing experiments such as those with chemical drugs. Because MSCs are living cells and our current understanding is limited, the process of MSCs in vivo is not very clear.

There were 17 psoriasis patients who completed UMSC infusions; 47.1% (8/17) had at least 40% improvement, 35.3% (6/17) had at least 75% improvement and 17.6% (3/17) had more than 90% improvement in PASI at 6 months. In addition, among the 6 patients who had an improvement of PASI 75, only 1 of the patients showed sustained improvement, while the others did not show significant improvement until the 6-month follow-up. We did not find a significant correlation between the therapeutic effect of UMSCs and the single infusion dose. Among the 6 patients who had at least 75% improvement in PASI, 3 of the patients received a single dose of 1.5 × 10^6^/kg, and the remaining 3 patients received a single dose of 2.0 × 10^6^/kg, 2.5 × 10^6^/kg and 3.0 × 10^6^/kg, respectively. These results are consistent with some reports. Ahmed Saad reported that in the MSC treatment of renovascular disease, the increases in cortical and total tissue perfusion and renal blood flow were of similar magnitude for different doses of MSC infused, a single infusion of MSCs 1.0 × 10^5^ or 2.5 × 10^5^ cells/kg.^23^ SO YOON AHN reported no significant difference between the low-dose group (5 × 10^6^ cells/kg) and the high-dose group (1 × 10^7^ cells/kg) in the efficacy of MSCs for severe intraventricular hemorrhage in preterm infants.^24^ Based on literature reports and animal experimental results, considering that the side effects of MSC infusion are mainly related to the single injection dose, the second stage mainly studies safety of different doses. Also, to compare efficacy, the total number of cells received by each patient during treatment was the same.

However, the role of UMSCs in vivo is not permanent. In the present study, recurrence occurred in 2 patients who had an improvement of PASI 90. They had disease relapse at 8 and 9 months, respectively, after the infusions. Additionally, some studies showed recurrence in SLE after UMSCT, approximately 18.9% (7/37) at the 9–12 month follow-up.^18^ The recurrence may be ascribed to the limited duration of UMSCs and the persistence of pathogenic factors. A repeated UMSC infusion is feasible and necessary after several months to avoid disease relapse. Notably, one patient who had an improvement of PASI 90 at the 6-month follow-up maintained one-year relapse-free status without using any other traditional drugs. Therefore, recruitment criteria and the factors related to recurrence need further research.

Interestingly, we found that UMSC treatment for psoriasis was more effective in females, with a response rate of 66.7%, than in males, with a response rate of 25%. We speculated that female psoriasis patients who showed a better response to UCMST may have an abnormality in the levels of sex hormones. MSCs may also affect the secretion of sex hormones by improving ovarian function in addition to regulating immune balance, thereby improving the pathology of psoriasis. Unfortunately, in the research protocol, we did not consider assessing sex hormone levels in patients. It is worth further phase 2 clinical study with a larger sample size to confirm whether there is indeed a gender difference in MSC treatment of psoriasis.

The pathogenesis of psoriasis is accompanied by the infiltration and overactivation of effector T cells, such as Th1, Th2, and Th17 cells, in psoriasis lesions, which leads to an impaired balance between Treg and effector T cells and an upregulation of proinflammatory cytokines.^25^ Th17 cells are a subset of T-lymphocytes expressing IL-17 that induce keratinocyte proliferation and other characteristics of psoriasis. Many approved biological agents that target the IL-17 pathway have achieved improved clinical outcomes. Tregs are a subpopulation of T lymphocytes that are committed to suppressing the immune response and maintaining immune homeostasis.^26^ Anti-TNF-α agents (especially etanercept),^27^ methotrexate^28^ and vitamin D^29^ treatments for psoriasis appear to increase Treg numbers in psoriasis. Several studies described an imbalance of the Treg to Th17 ratio in psoriasis.^30,31^ It was reported that the percentage of CD4^+^CD25^+^Foxp3^+^ (Tregs) was increased and that the percentage of CD4^+^IL-17^+^ (Th17) cells was decreased in rheumatoid arthritis after MSC treatment.^32^ Junji Xu et al. reported that MSC infusion directed T cells toward Treg and Th2 cells while suppressing Th17 responses and alleviating Sjogren syndrome (SS) symptoms.^13^ Collectively, these studies suggest that MSCs play an important role in regulating immune homeostasis. Consistently, we observed an increase in Tregs and a decrease in Th17 cells and the associated cytokine IL-17 in the response group after UMSCT. Our results suggested that UMSCs may play a therapeutic role in psoriasis through the Treg-Th17-cell axis, which is consistent with our previous study in mouse models of psoriasis.^15^ Previous studies have found that MSCs have different regulatory effects on naive, memory and effector T cells in autoimmune diseases.^33,34^ In our study, we observed a significant decrease in CD4 naive T cells and an increase in effector T cells, such as CD4 TCMs, CD4 memory T cells and Tregs, in responsive patients after UMSCT, which suggests that MSC infusions may promote T-cell differentiation into Tregs and memory T cells.

In recent years, the role of B cells in the development of autoimmune diseases has attracted much attention. F. Facciotti identified a previously uncharacterized population of extrafollicular B helper T cells, which produced IL-10 and could play a prominent pathogenic role in SLE.^35^ Li reported that the abnormal increases in GM-CSF^+^B-cell frequencies and responses in patients with MS, their enhanced capacity to activate proinflammatory myeloid cells, and the observations from B-cell depletion therapy in patients all point to a pathogenic role for the effector B-cell subset in MS.^36^ However, the relationship between B cells and psoriasis is still unclear. Therefore, we analyzed the differences in B-cell subsets between patients and HCs. We did not observe significant differences in the percentages and numbers of B cells and subsets between HC and psoriasis patients or psoriasis pre- and post-UMSCT (data not shown).

In our study, UMSCT was effective in only some of the patients. To analyze the possible reason and try to find the predictive indicators related to the efficacy of UMSCT, we compared the baseline levels of immune cells and proinflammatory factors between responders and nonresponders. The results showed that the baseline levels of CD4+ TCM cells, Treg cells, and Treg/Th17 cells in PB in responsive patients were significantly lower than those in nonresponsive patients. Because no studies have shown that CD4+ TCM is related to the pathogenesis of psoriasis, no significant difference in the baseline level of Th17 between responsive and nonresponsive patients was found. Therefore, we assumed that a low level of Treg cells predicted a better response to UMSCs and that it may be an effective predictor of UMSCT in patients with psoriasis.

In general, intravenous infusions of UMSCs in the treatment of psoriasis were safe and partly effective, especially in females. In addition, lower Treg levels may be used as a predictor of the effectiveness of UMSC transplantation. However, these findings need to be further expanded for verification in a larger and well-controlled prospective clinical trial.

## Materials and methods

### All these ethics statements

The clinical trial was performed according to the Declaration of Helsinki. This study was approved by the National Health Commission of the People’s Republic of China and the Ethics Committee of Xiangya Hospital Central South University (No. 201708085. Full names of the Ethics committees are: Guanghua Lei, Tao Yin, Shifang Peng, Hong Jiang, Yaolei Wang, Meilian Yang, Hua Tan). All patients provided written informed consent. Human blood and urine samples from patients were collected by Xiangya hospital with consent from all patients.

### Study design

This study was a single-center, open-label, single-arm study conducted at Xiangya Hospital Central South University in China. The clinical trial was registered with ClinicalTrials.gov (registration number: NCT03765957).

The primary objective of this study was to assess the safety and efficacy of UMSCs in treating psoriasis. The clinical trial was conducted in two stages. It should be noted that in the first stage, a randomized controlled trial was designed to compare the safety and efficacy of UMSCs and MTX, but the researchers canceled the MTX group because of the slow enrollment and poor compliance and changed the protocol to an open label single-arm study to evaluate the safety, tolerance, and efficacy of UMSCs in the treatment of psoriasis following the advice from the Ethics and Academic Committee. The first stage was from Mar to Sep 2019, and the patients were screened and received UMSC infusions once every 2 weeks in a single dose of 1.5 × 10^6^/kg, 4 times as a course of treatment, followed up at 15 days, 30 days, 45 days, 2 months, 3 months and 6 months after treatment. In the second stage, from Sep 2019 to Aug 2020, the researchers set up 4 UMSC groups of escalating doses (1.5, 2.0, 2.5, 3.0 × 10^6^/kg). Patients were screened and randomly assigned to MSC treatment group A, B, C and D at a ratio of 1:1:1:1. The dose of treatment group A was 1.5 × 10^6^/kg, The dose of treatment group B was 2.0 × 10^6^/kg, The dose of treatment group C was 2.5 × 10^6^/kg, The dose of treatment group D was 3.0 × 10^6^/kg. Patients in group A and B were intravenously infused with UMSCs once every 15 days, 4 times as a course of treatment, followed up at 15 days, 30 days, 45 days, 2 months, 3 months and 6 months after treatment. Patients in group C and D were intravenously infused with UMSCs once every 30 days, 2 times as a course of treatment, followed up at 15 days, 30 days, 45 days, 2 months, 3 months and 6 months after treatment. Due to the lack of dose standards and long-term safety monitoring for UMSC in the world, the high concentration group (2.5, 3.0 × 10^6^/kg) extended the injection interval to ensure the same total number of cells injected in the patients, following the principle of caution and patient safety first. See Fig. 1 for the details of the study design. The statistics of patients in stages 1 and 2 were analyzed together. The 4 groups of stage 2 did not show significant differences in safety and efficacy, so the researchers explored these statistics together.

### The patients

Eligible patients were 18–65 years old with stable (>6 months) psoriasis vulgaris and had not received stem cell therapy in the past 6 months. Body-surface area (BSA) involvement >10%, Physician Global Assessment (PGA) score >3 and Psoriasis Area Severity Index (PASI) score >12 at screening and baseline. At the second stage, the inclusion criteria of the PASI score were modified to >8 at screening and baseline for better enrollment according to the advice from the Ethics and Academic Committee. In stage 1, it was found that the efficacy of UMSC on psoriasis was not ideal and did not reach the height of the efficacy of the current targeted treatment of psoriasis, so in stage 2, to make a more accurate target population positioning for the future UMSC treatment of psoriasis, the researchers reduced severity requirements for psoriasis patients. Additional eligibility criteria were an inadequate response or intolerance to at least one conventional systemic agent (i.e., acitretin, cyclosporin A, biological agent or ultraviolet phototherapy). Patients were ineligible if they had received systemic medications in the previous 1 month, used external medications in the previous 2 weeks or participated in other clinical studies in the past 3 months. See Supplementary Table 2 for additional exclusion criteria.

### Randomization

A random sequence was generated using SPSS with a random seed. In the second stage, patients were assigned to the four groups according to the rank of their random numbers. An investigator at Xiangya Hospital generated the randomization scheme.

### Outcomes

The primary outcome was the number of patients achieving PASI ≥ 75% (PASI 75) improvement at month six. The secondary outcome was the number of patients achieving a PGA of 0 or 1 at month six. Each patient returned for follow-up at day 0 (D0), D15, D30, D45, and month 2 (M2), M3 and M6 after UMSCT (Fig. 2). Evaluations performed at these follow-up visits included a physical examination, determination of PASI score, BSA and PGA analysis, hematology, and clinical biochemistry analysis. Adverse events and their severity were assessed and recorded throughout the study. Efficacy evaluations included the PASI and the PGA. The PASI score is based on the extent of psoriatic involvement of the body-surface area on the head, trunk, arms, and legs and the severity of scale formation, erythema, and plaque induration in each region of the body. The PGA score reflects the overall status of psoriatic lesions (induration, erythema, and scaling). Safety was evaluated by assessing adverse events and routine hematologic and laboratory values (such as myocardial zymogram and clinical biochemistry analysis). Further details are provided in Supplementary Table 3.

### GMP manufacturing and characterization of UMSCs

The clinical-grade UMSCs were supplied, free of charge, by the National Engineering Research Center of Human Stem Cells and The Guangxiu Tech Biotechnology Co. Preparation was completed in a GMP laboratory. Master cell bank at passage 1 and working cell bank at passage 4 were identified by the criteria suggested by the International Society for Cellular Therapy (ISCT): (1) plastic-adherent in standard culture conditions using tissue culture flasks; (2) ≥95% of the MSC population express CD105, CD73 and CD90 and lack expression of (≤2% positive) of CD45, CD34, CD11b, CD19 and HLA-DR as measured by flow cytometry (BD Accuri C6, USA); (3)differentiation potential to osteoblasts, adipocytes and chondroblasts under standard in vitro differentiating conditions. Moreover, each batch of master cell bank and working cell bank was tested for sterility (bacterial, fungal, mycoplasma and endotoxin), human virus (HAV, HBV, HCV, TP, CMV, EBV, B19, HSV-1, HSV-2, HHV-6, HIV, and HTLV), porcine parvovirus, bovine diarrhea virus, mycobacterium tuberculosis, proliferation ability, cell cycle, short tandem repeat (STR), G-banding karyotype analysis, tumorigenicity, immunomodulatory capacity, telomere length and telomerase activity. In preparation for infusion, frozen UMSCs at passage 4 were quickly thawed, washed, and suspended in normal saline supplemented with human serum albumin. For each MSC product, cell counts and viability were examined using trypan blue staining by a cell counter (Countstar, China) as product release criteria. The numbers of cells depend on the patient’s body weight, which is defined in the clinical protocol.

### Analysis of immune cell subsets

A large panel of leukocyte markers was assayed with flow cytometry to quantify multiple immune cell subpopulations in the patients’ PB at visit 0 (Pre-UMSCT) and visit 8 (Post-UMSCT). Heparinized PB samples were collected from 17 psoriasis patients and incubated with antibodies at room temperature in the dark for 20 min. Red blood cells were lysed using red blood cell lysis buffer (BD Biosciences, USA). After washing with DPBS buffer, resuspended cells were detected on a FACS Canto PLUS Flow cytometer (BD Biosciences) and analyzed with FlowJo software. Information on the antibodies is detailed in Supplementary Table 4.

To detect T-cell subpopulations, PBMCs from patients were isolated with standard Ficoll density gradient centrifugation techniques by Ficoll-Paque Plus (Tianjin Haoyang, China) (1·077 g/ml) according to the manufacturer’s instructions. PBMCs were stimulated for 4–6 h with Cell Activation Cocktail (Biolegend, USA) in the presence of Monensin Solution (Biolegend). Intracellular staining was performed using the Human Th1/Th2/Th17 Phenotyping Kit (BD Biosciences) according to the manufacturer’s instructions to identify human CD4, IFN-γ (for Th1), IL-4 (for Th2), and IL-17A (for Th17). After suspension with 1 ml cold BD Cytofix^TM^ Fixation Buffer (provided in the kit) and incubation for 20 min at room temperature, the cells were permeabilized and stained with the cocktail (provided in the kit). After washing with DPBS, resuspended cells were acquired using a FACS Canto PLUS Flow cytometer (BD Biosciences) and analyzed with FlowJo software.

### Cytokine detection

Serum was collected at Visits V0 (Pre-UMSCT) and Visits 8 (Post-UMSCT) in psoriasis patients and stored at −80 °C until analysis. The levels of TNF-α, IL-6, IL-1β, and IL-17 in serum were analyzed with ELISA kits (R&D Systems, USA).

### Statistical analysis

Data were analyzed and collected up to August 2020. Patients were censored at the time of the last follow-up. Descriptive data are presented as the mean (standard deviation [SD]), median (interquartile range [IQR]), and number (%). To test differences between pre- and post-UMSCT, matched samples t- test was used for normally distributed continuous data and Wilcoxon matched-pairs signed-rank test for non-normally distributed continuous data. To test differences between psoriasis patients and healthy controls or female and male, two independent sample t-test was used for normally distributed continuous data and Wilcoxon (Mann-Whitney) test for non-normally distributed continuous data. All statistical analyses were done with SPSS version 20·0 statistical software (IBM SPSS). All P values were two-sided, and *P* < 0.05 was considered statistically significant.

## Supplementary information

Supplementary Materials for Human umbilical cord mesenchymal stem cells for psoriasis: A phase 1/2a, single-arm study

## Data Availability

The datasets generated during and/or analyzed during the current study are available from the corresponding author on reasonable request.
