# Supplementary material for: Human umbilical cord mesenchymal stem cells for psoriasis: a phase 1/2a, single-arm study
Source: Signal Transduct Target Ther. 2022 Aug 5;7:263. doi: 10.1038/s41392-022-01059-y (PMC9352692; doi:10.1038/s41392-022-01059-y)
Supplement: Supplementary file 1 — Supplementary Materials for Human umbilical cord mesenchymal stem cells for psoriasis: A phase 1/2a, single-arm study [file 41392_2022_1059_MOESM1_ESM.docx]

Supplementary Materials for

Human umbilical cord mesenchymal stem cells for psoriasis: A phase 1/2a, single-arm study

*Lamei Cheng^13*^ ，Siqi Wang^3*^, Cong Peng^24*^, Xiao Zou^3^, Chao Yang^3^, Hua Mei^3^, Chuang Li^3^, Xian Su^3^, Na Xiao^13^, Qi Ouyang^13^, Mi Zhang^24^, Qiaolin Wang^24^, Yan Luo^24^，Minxue Shen^24^, Qun Qin^6^, Honglin Wang^7^, Wu Zhu^24^, Guangxiu Lu^135^,Ge Lin^135#^, Yehong Kuang^24#^, Xiang Chen^24#^*

Correspondence to: Xiang Chen([chengxiangck@126.com](mailto:chengxiangck@126.com)); Yehong Kuang ([yh_927@126.com](mailto:yh_927@126.com)); Ge Lin ([linggf@hotmail.com](mailto:linggf@hotmail.com)).

**This PDF file includes:**

Figures. S1 to S5

Tables S1 to S4


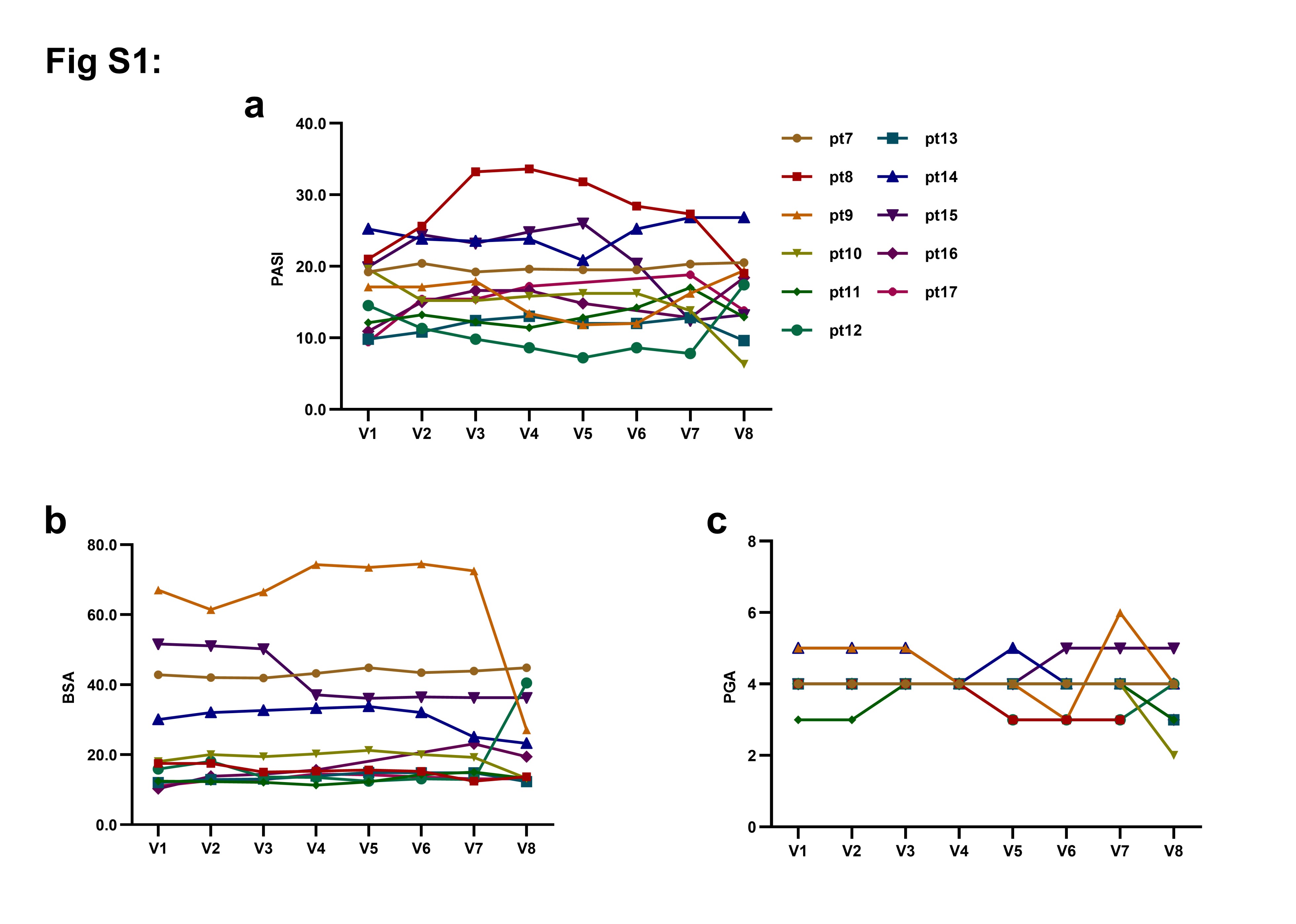


Fig S1 The figure showed three outcome measures of the 11 nonresponders including PASI score, BSA and PGA. (a) PASI score; (b) BSA; (c) PGA.


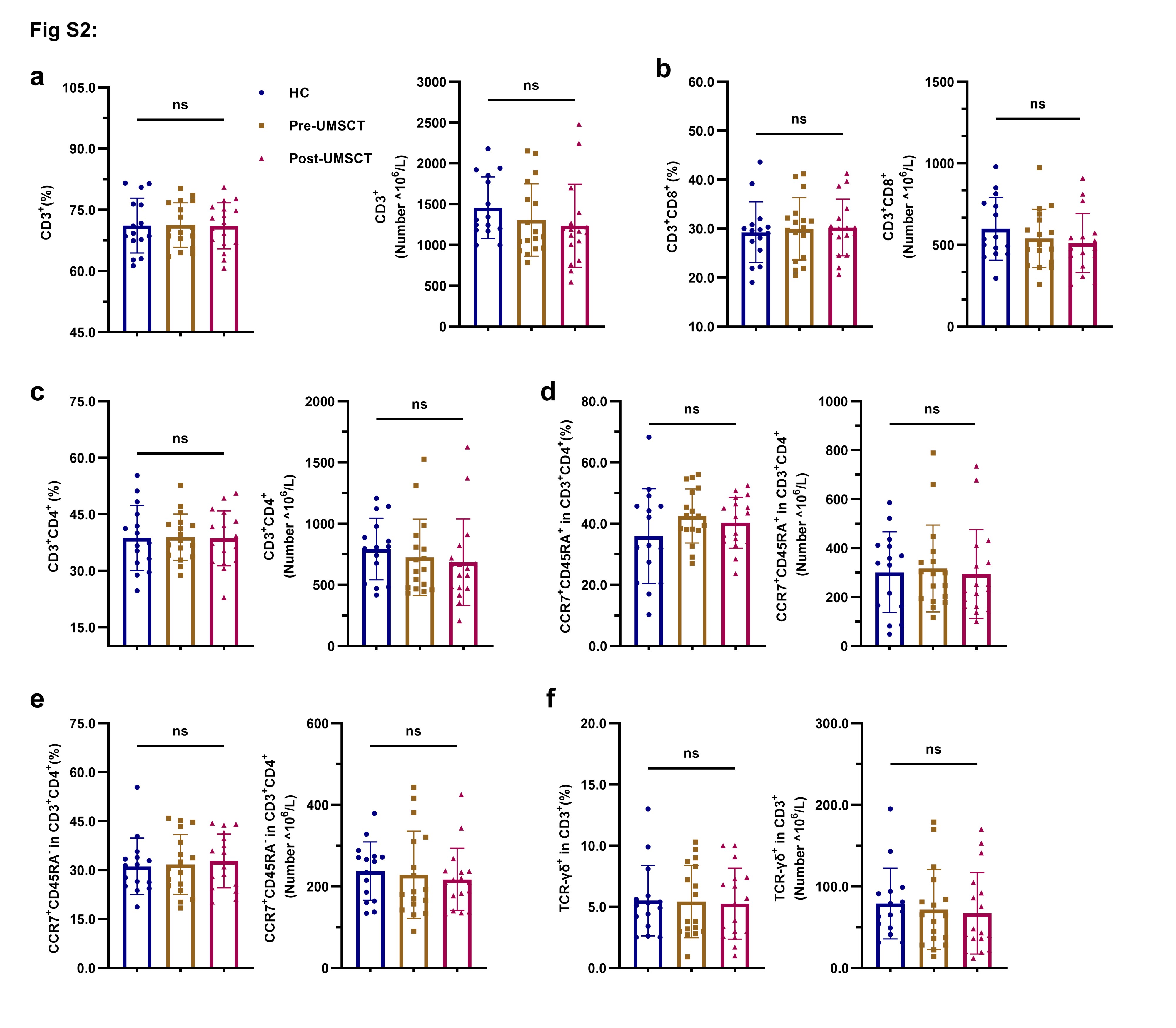


FigS2. a-f. Frequencies and numbers of CD3^+^、CD3^+^CD8^+^、CD3^+^CD4^+^、CD4^+^naïve T、 CD4^+^TCM and γδ T cells in PB in HC and psoriasis patients pre- and post- UMSCT (HC, n=15; Patients, n=17; ns, no significant, VS HC and VS Pre-UMSCT).


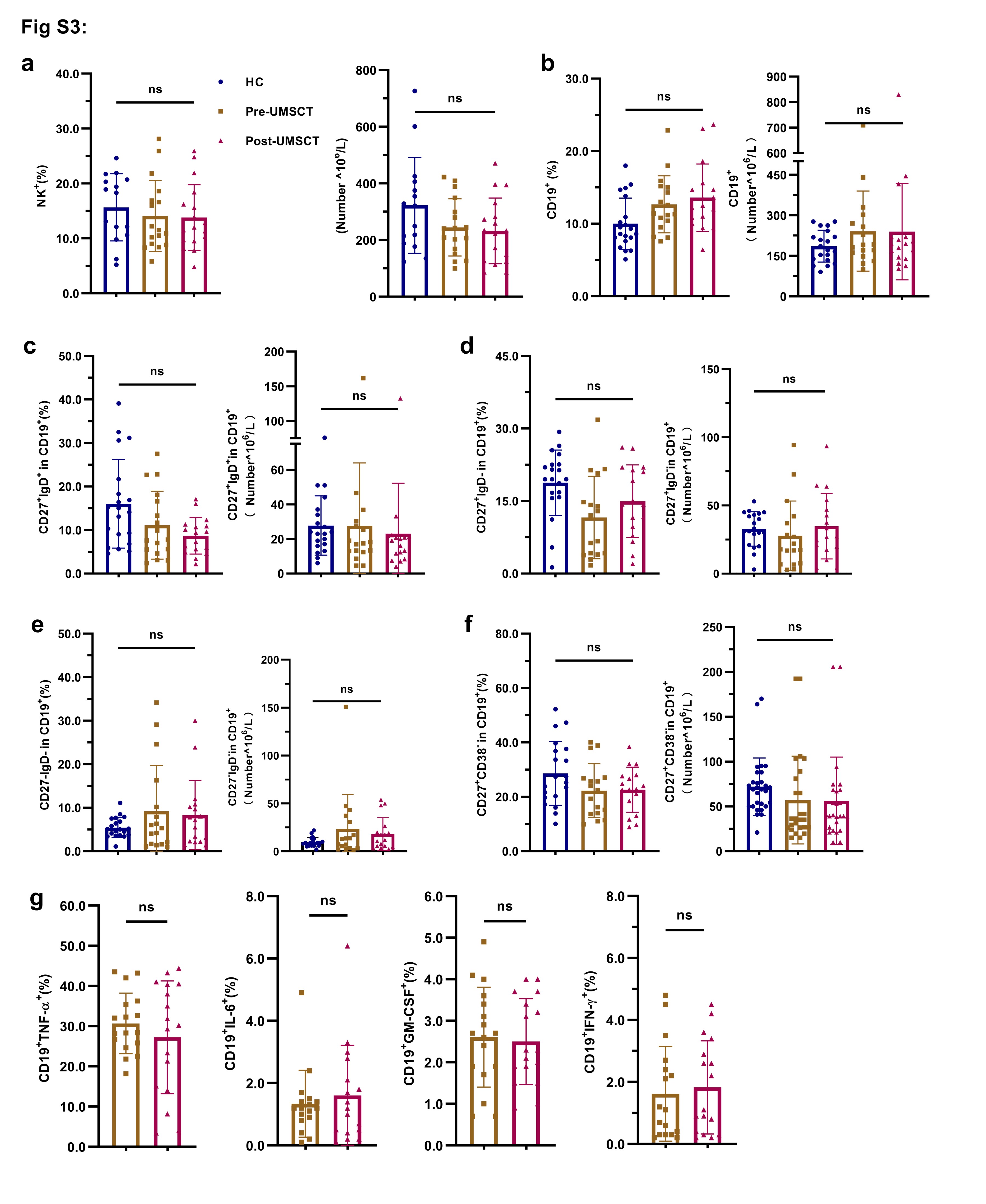


FigS3. a-f. Frequencies and numbers of NK^+^、CD19^+^、unswitched memory B、switched memory B、DN B and memory B cells in PB in HC and psoriasis patients pre- and post-UMSCT (HC, n=15; Patients, n=17; ns, no significant, VS HC and VS Pre-UMSCT); g: Frequencies of CD19^+^TNF-α^+^、CD19^+^IL-6、CD19^+^GM-CSF^+^、CD19^+^IFN-γ^+^ cells in PB in psoriasis patients pre- and post UMSCT (Patients, n=17; ns, no significant, VS Pre-UMSCT).


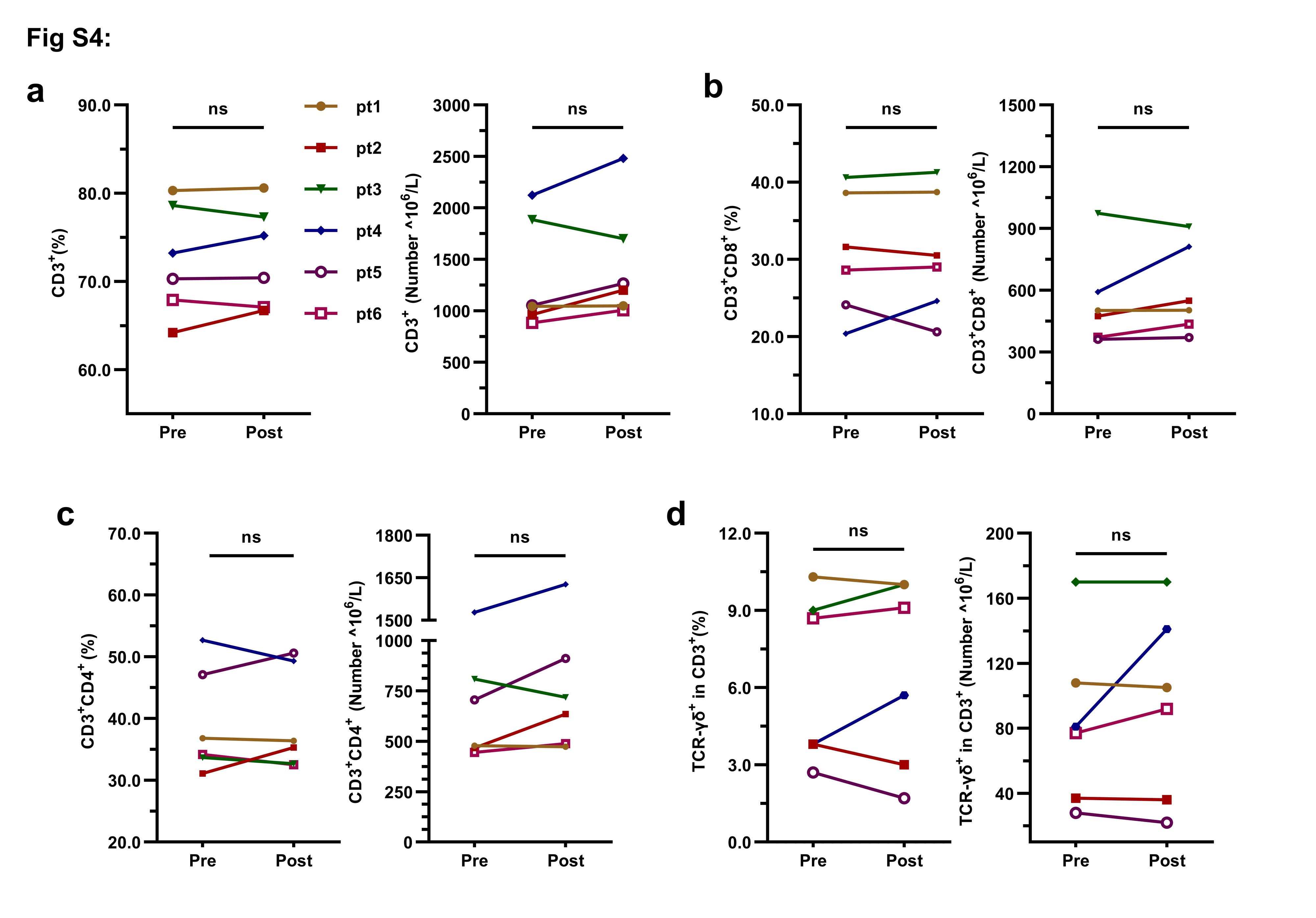


FigS4. a-d: The frequencies and numbers of CD3^+^、CD3^+^CD8^+^、CD3^+^CD4^+^and γδ T cell in PB in response group.(n=6, ns, no significant)


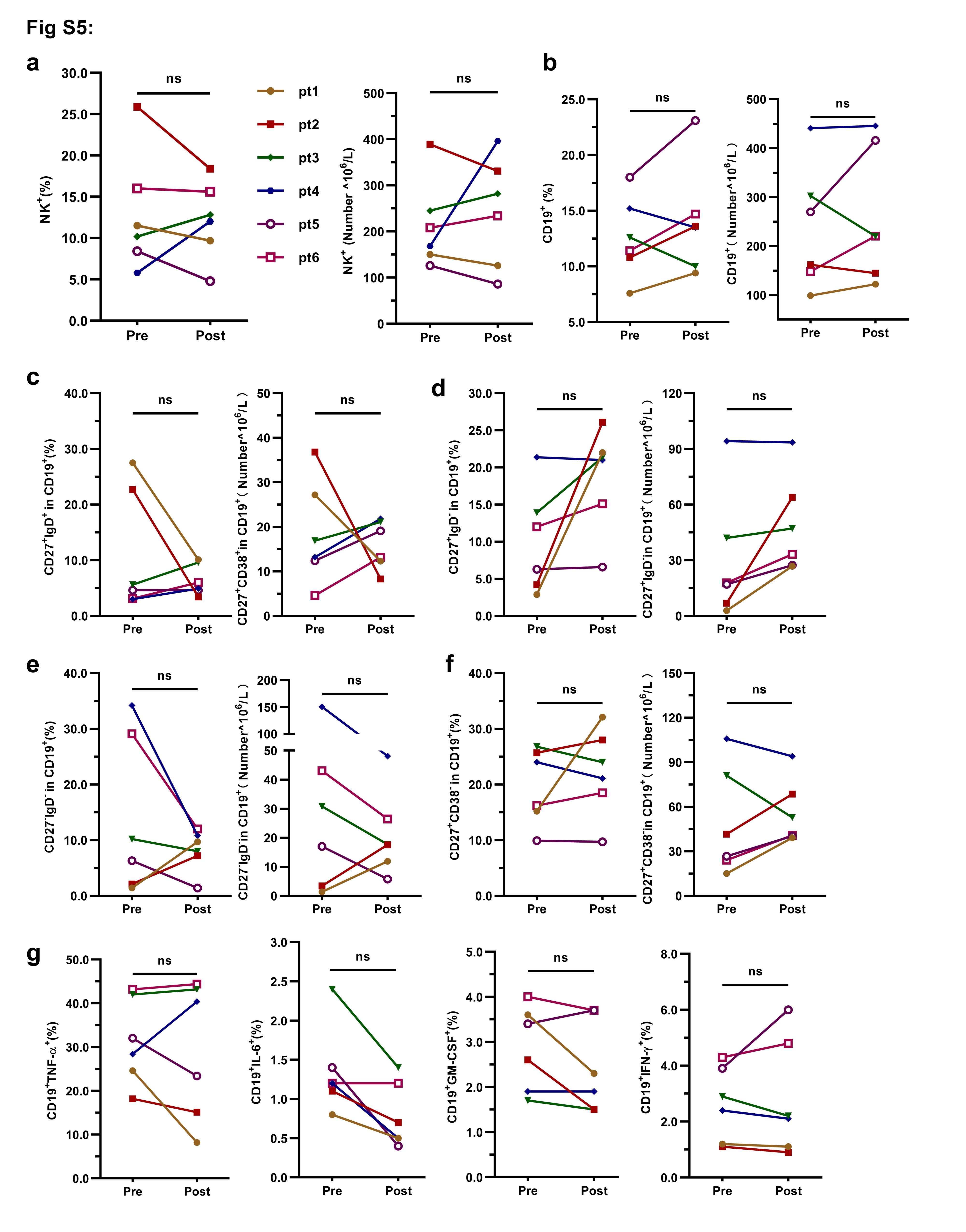


FigS5. a-f. Frequencies and numbers of NK^+^、CD19^+^、unswitched memory B、switched memory B、DN B and memory B cells in PB in response group. (n=6, ns, no significant); g: Frequencies of CD19^+^TNF-α^+^、CD19^+^IL-6^+^、CD19^+^GM-CSF^+^、CD19^+^IFN-γ^+^ cells in PB in response group. (n=6, ns, no significant).

**Table S1: Descriptions and phenotypes of immune cell populations.**

Cell subsets

| Description | Phenotype | Parent population (%) |
| --- | --- | --- |
| T-cells | CD3+ | Lymphocytes |
| CD4+ T cells | CD4+ | Lymphocytes |
| CD8+ T cells | CD8+ | Lymphocytes |
| CD8+ T helper cells | CD3+ CD8+ | CD3+ T-cells |
| CD4+ T helper cells | CD3+ CD4+ | CD3+ T-cells |
| TCR γδ+ T-cells | TCR-γδ+ in CD3+ | CD3+ T-cells |
| CLA+ T cells | CLA+ in CD3+ | CD3+ T-cells |
| memory T-cells | CD45RO+ in CD3+ | CD3+ T-cells |
| memory CD4+ T-cells | CD45RO+ in CD3+CD4+ | CD4+ T-cells |
| memory CD8+ T-cells | CD45RO+ in CD3+CD8+ | CD8+ T-cells |
| Treg | CD25+CD127-/low in CD3+CD4+ | CD4+ T-cells |
| CD4+ naïve T cells | CCR7+CD45RA+ in CD3+CD4+ | CD4+ T-cells |
| Central Memory CD4+ T_CM_-cells | CCR7+CD45RA- in CD3+CD4+ | CD4+ T-cells |
| Effector Memory CD4+ T_EM_-cells | CCR7-CD45RA- in CD3+CD4+ | CD4+ T-cells |
| Effector Memory CD45RA+ CD4+ TEMRA-cells | CCR7-CD45RA+ in CD3+CD4+ | CD4+ T-cells |
| CD8+ naïve T cells | CCR7+CD45RA+ in CD3+CD8+ | CD8+ T-cells |
| Central Memory CD8+ T_CM_-cells | CCR7+CD45RA- in CD3+CD8+ | CD8+ T-cells |
| Effector Memory CD8+ T_EM_-cells | CCR7-CD45RA- in CD3+CD8+ | CD8+ T-cells |
| Effector Memory CD45RA+ CD8+ TEMRA-cells | CCR7-CD45RA+ in CD3+CD8+ | CD8+ T-cells |
| NK-cells | CD3- CD56+ | Lymphocytes |
| B-cells | CD3-CD19+ | Lymphocytes |
| Transitional B cells | CD24hiCD38hi in CD19+ | B-cells |
| primarily memory B cells | CD24hiCD38- in CD19+ | B-cells |
| primarily mature B cells | CD24intCD38int in CD19+ | B-cells |
| Mature B cells | CD21+ in CD19+ | B-cells |
| Active B cells | CD21-/lowCD38-/low in CD19+ | B-cells |
| Non-switched B-cells | CD27+IgD+ in CD19+ | B-cells |
| Class-switched B-cells | CD27+IgD- in CD19+ | B-cells |
| DN B cells | CD27-IgD- in CD19+ | B-cells |
| Naïve B cells | CD27-IgD+ in CD19+ | B-cells |
| Memory B cells | CD27+CD38- in CD19+ | B-cells |
| Plasmablasts | CD27+CD38+ in CD19+ | B-cells |

**Table S2: Patient Clinical Study Eligibility Criteria.**

| **Main (but not exhaustive) Inclusion Criteria:** |
| --- |
| 1. patients' informed consent, voluntary participation, and signed informed consent form. 2. the age is 18 to 65 years old, the sex is not limited, and the body mass index (BMI) is between 18.5 and 35, including the boundary value. 3. patients with psoriasis vulgaris with a course of more than 6 months were unsatisfied or unable to tolerate at least one systematic treatment (acitretin, CsA, biological agents, ultraviolet phototherapy, etc.). The BSA score of psoriasis vulgaris was > 10%, PGA score > 3 and PASI score > 12 at screening and baseline. (PASI score > 8 at screening and baseline for stage two) 4. did not receive stem cell therapy in recent 6 months. |
| **Main (but not exhaustive) Exclusion Criteria:** |
| 1. Hemogram: total white blood cell count < 3.5×10^9^/L, platelet count < 100×10^9^/L, hemoglobin < 100g/L, heart, kidney and liver insufficiency, total bilirubin or serum creatinine > 1.5 times of normal upper limit, glutamic oxaloacetic transaminase or glutamic pyruvic transaminase > 2 times of normal upper limit, serum HBs antigen positive, HIV antibody positive, syphilis antibody positive, HCV positive. 2. complicated with pneumonia, severe infection, malignant lesions (such as tumor); patients with severe systemic or organic diseases, severe mental illness and cognitive impairment. 3. received systemic drug treatment in the past month and topical drug treatment in the past 2 weeks. 4. have a history of severe allergic reactions or are allergic to two or more kinds of food or drugs. 5. pregnant or participated in other clinical studies in recent 3 months, or any other reason that the researchers considered not to participate in the trial. |

| **Table S3: Summary of the Patient Assessment during the Trial** | | | | | | | | |  |
| --- | --- | --- | --- | --- | --- | --- | --- | --- | --- |
| **Experimental stage** | **Screen** | **Therpy** | | | | **follow-up** | | |  |
|  | **V1** | **V2** | **V3** | **V4** | **V5** | **V6** | **V7** | **V8** |  |
| **Time points** | **14 ~0d** | **D0 ±2d** | **D15 ±2d** | **D30 ±2d** | **D45 ±2d** | **M2 ±2d** | **M3 ±7d** | **M6 ±7d** |  |
| **Patient study inclusion** |  |  |  |  |  |  |  |  |  |
| Informed consent | X |  |  |  |  |  |  |  |  |
| Selection / exclusion criteria | X |  |  |  |  |  |  |  |  |
| Demographic data, medical history | X |  |  |  |  |  |  |  |  |
| Urinary pregnancy immunity test **a** | X |  |  |  |  |  |  |  |  |
| General record | X |  | X | X | X | X | X | X |  |
| Vital signs | X | X | X | X | X | X | X | X |  |
| Four items of blood transfusion | X |  |  |  |  |  |  |  |  |
| Complete set of virus | X |  |  |  |  |  |  |  |  |
| PASI / BSA score | X |  | X | X | X | X | X | X |  |
| sPGA | X |  | X | X | X | X | X | X |  |
| Scale **b** | X |  | X | X | X | X | X | X |  |
| **Clinical assessment** |  |  |  |  |  |  |  |  |  |
| Blood routine | X |  | X | X |  | X |  | X |  |
| Urine routine | X |  |  | X |  | X |  | X |  |
| Liver and kidney function | X |  |  | X |  | X |  | X |  |
| blood glucose and lipids | X |  |  | X |  | X |  | X |  |
| E7A | X |  |  | X |  | X |  | X |  |
| IgG 、 IgM 、 IgA | X |  |  | X |  | X |  | X |  |
| ESR 、 CRP | X |  |  | X |  | X |  | X |  |
| Electrocardiogram | X |  |  | X |  | X |  | X |  |
| Chest film | X |  |  |  |  | X |  | X |  |
| Tumor marker **c** | X |  |  |  |  | X |  | X |  |
| TB check | X |  |  | X |  | X |  | X |  |
| Myocardial zymogram | X |  |  | X |  | X |  | X |  |
| Combined use of drugs | X | X |  | X | X | X | X | X |  |
| Cell therapy |  | X |  | X | X |  |  |  |  |
| Adverse events |  | X |  | X | X | X | X | X |  |
| Case summary |  |  |  |  |  |  |  |  |  |
| a. urinary pregnancy immunity test (female) is used only for women who may be pregnant for blood or urine pregnancy tests. Postmenopausal women (who had not had menstruation for more than one year before signing the informed consent form) or who had undergone surgical sterilization were excluded.  b.including dermatology quality of life scale, depression scale, suicide scale (DLQI, PDI, Sf-36, Scalpdex, Skindex 29&16). c.tumor markers includes: AFP, CEA, lung cancer, CA50, CA125, CA199, male patients plus tPSA, fPSA. Abbreviations: V, trial visit; d, day; m, month(s). | | | | | | | | |  |
|  |  |  |  |  |  |  |  |  |  |
|  |  |  |  |  |  |  |  |  |  |
|  |  |  |  |  |  |  |  |  |  |
|  |  |  |  |  |  |  |  |  |  |

**Table S4: mAb of immune cell populations**

| Description | Manufactor | Fluorescein | Clone |
| --- | --- | --- | --- |
| CD3 | BD | FITC | SK7 |
| CD4 | BD | APC | SK3 |
| CD8 | BD | PE | SK1 |
| TCR γδ | Biolegend | APC | B1 |
| CLA+ | Biolegend | Percp/cy5.5 | HECA-452 |
| CD45RO | Biolegend | PE | UCHL1 |
| CD25 | Biolegend | APC | M-A251 |
| CD127 | Biolegend | PE/cy7 | A019D5 |
| CCR7 | Biolegend | Percp/cy5.5 | G043H7 |
| CD45RA | Biolegend | PE | H1100 |
| CD56 | BD | PE | NCAM16.2 |
| CD19 | BD | APC-H7 | SJ25C1 |
| CD19 | Biolegend | PE | HIB19 |
| CD24 | BD | BB700 | ML5 |
| CD38 | BD | BV421 | HIT2 |
| CD21 | BD | PE/cy7 | B-Ly4 |
| IgD | BD | BB515 | IA6-2 |
| IL-17 | Biolegend | Percp/cy5.5 | BL168 |
| IL-4 | Biolegend | APC | 8D4-8 |
| TNF-α | Biolegend | Percp/cy5.5 | MAb11 |
| IFN-γ | Biolegend | FITC | B27 |
| IL-6 | Biolegend | FITC | MQ2-13A5 |
| GM-CSF | Biolegend | Percp/cy5.5 | BVD2-21C11 |
